# Supplementary material for: Use of a Calving Blind That Imitates a Natural Environment
Source: Animals (Basel). 2024 Apr 13;14(8):1171. doi: 10.3390/ani14081171 (PMC11047498; doi:10.3390/ani14081171)
Supplement: Supplementary file 1 [file animals-14-01171-s001.zip › animals-2937478-SI/Supplementary File S1.pdf]

Supplementary File S1. The number of agonistic bouts (head butt, chase, and displacement from a blind), allogrooming bouts, and allogrooming duration (s) for each cow included in the focal cow analysis during the 5 h prior to calving. The cow could have been the actor or receiver for each interaction summarized.

Cow ID	Agonistic bouts	Allogrooming bouts	Allogrooming duration (s)
1240	2	0	
1251	9	0	
1272	0	1	23
1339	3	0	
1340	5	6	252
1347	3	0	
1351	4	0	
1354	2	0	
1357	7	0	
1367	17	0	
1391	21	0	
1400	11	0	
1406	1	18	794
1414	8	0	
1418	8	0	
1422	19	0	
1433	13	0	
1442	9	0	
1443	2	0	
1446	4	1	62
1455	13	0	
1457	5	0	
1458	5	0	
1459	11	0	
1462	21	0	
1463	12	0	
1467	14	2	89
1469	5	2	112
1471	12	0	
1477	1	0	
