# Supplementary material for: Use of a Calving Blind That Imitates a Natural Environment
Source: Animals (Basel). 2024 Apr 13;14(8):1171. doi: 10.3390/ani14081171 (PMC11047498; doi:10.3390/ani14081171)
Supplement: Supplementary file 1 [file animals-14-01171-s001.zip › animals-2937478-SI/Supplementary File S2.pdf]

Supplementary File S2. The descriptive summary of the number of times a cow was observed in a blind during the 12 h before calving and the same 12 h period on day 7 prior to calving. Scan sampling was conducted to record if the cow was in or out of a blind every 10 min for a 12 h period. It is also defined if the cow gave birth in the blind.

	# of times a cow was observed in a blind in a 12 h period		
Cow ID	7 d before calving	Day of calving	Calved in blind
1240	0	9	Yes
1251	0	43	Yes
1272	52	23	Yes
1339	2	2	No
1340	23	27	No
1347	13	20	Yes
1351	0	10	Yes
1354	0	0	No
1357	1	20	Yes
1367	2	35	Yes
1391	15	15	No
1400	7	29	Yes
1406	1	0	No
1414	1	12	No
1418	8	54	Yes
1422	2	11	No
1433	12	35	Yes
1442	8	2	No
1443	8	11	No
1446	38	22	Yes
1455	0	16	No
1457	21	23	Yes
1458	3	6	Yes
1459	0	12	Yes
1462	0	5	No
1463	0	7	No
1467	0	3	No
1468	0	11	Yes
1469	12	8	No
1471	0	11	No
1477	13	31	No
